# The Effect of *Santolina chamaecyparissus* and *Tagetes patula* Essential Oils on Biochemical Markers of Oxidative Stress in Aphids

**DOI:** 10.3390/insects12040360

**Published:** 2021-04-17

**Authors:** Paweł Czerniewicz, Grzegorz Chrzanowski

**Affiliations:** 1 Institute of Biological Sciences, Faculty of Exact and Natural Sciences, Siedlce University of Natural Sciences and Humanities, Prusa 14, 08-110 Siedlce, Poland; 2 Department of Biotechnology, Institute of Biology and Biotechnology, University of Rzeszow, Zelwerowicza 8B, 35-601 Rzeszow, Poland; gchrzanowski@ur.edu.pl

**Keywords:** botanical insecticide, toxicity, ROS, TBARS, *Myzus persicae*, *Rhopalosiphum padi*

## Abstract

**Simple Summary:**

Due to numerous side effects associated with extensive use of chemical insecticides, there is a need to develop eco-friendly alternative methods for insect pest control. One of these alternatives may be the use of essential oils (EOs). An important aspect of the efficient and safe application of EOs in plant protection is the elucidation of their toxicity mechanisms towards target pests. The present study aimed to determine the effects of *Santolina chamaecyparissus* (L.) and *Tagetes patula* (L.) EOs on development and physiology of two aphid species with different feeding specializations. *Myzus persicae* (Sulzer) is a broad generalist and *Rhopalosiphum padi* (L.) is a grass specialist. Exposure to the tested EOs limited the aphids’ development and led to induction of oxidative stress within their tissues. Analysis of the physiological parameters also showed that the oligophagous *R. padi* was more sensitive to EO treatment than the highly polyphagous *M. persicae*. The results suggest that the tested EOs can affect important biochemical processes within aphid tissues and have potential as eco-friendly aphicides.

**Abstract:**

This study investigated the toxicity of essential oils (EOs) from *Santolina chamaecyparissus* (L.) and *Tagetes patula* (L.) towards the green peach aphid *Myzus persicae* (Sulzer) and the bird cherry-oat aphid *Rhopalosiphum padi* (L.). The effects of the EOs on aphid population parameters and levels of biochemical markers of oxidative stress within insect tissues were analyzed. In laboratory bioassays, application of the studied EOs at sublethal concentrations reduced daily fecundity and led to a decrease in the intrinsic rate of natural increase in both aphid species. Treatment with EOs also induced generation of reactive oxygen species (ROS) within aphid tissues. The highest levels of superoxide anion and hydrogen peroxide were noted after 24 and 48 h of exposure. Moreover, a significant increase in lipid peroxidation was shown in treated aphids, especially between 48 and 72 h after exposure. The increase was more pronounced after treatment with the essential oil of *S. chamaecyparissus*, which also exhibited higher aphicidal activity in toxicity tests. The activities of antioxidant enzymes—superoxide dismutase (SOD) and catalase (CAT)—were significantly elevated in both aphid species in response to the tested EOs. The obtained results suggest that oxidative stress evoked by treatment with the studied EOs may be an important factor determining their toxicity towards aphids.

## 1. Introduction

Aphids are important insect pests of many agricultural and horticultural crops. During feeding they extract phloem sap, which contains essential nutrients, reducing the vigor and productivity of the plant. In addition, aphids may cause significant damage to the crops by transmission of numerous plant viruses [1]. The green peach aphid, *Myzus persicae* (Sulzer), is a highly polyphagous aphid that feeds on hundreds of host plants from more than 40 plant families, causing huge economic losses in a wide range of crops. In contrast, the bird cherry-oat aphid *Rhopalosiphum padi* (L.) possesses a distinctly narrower range of host plants, mainly from the Poaceae family, and in particular causes serious damage to cereal crops [2,3]. It is worth noting that aphids infesting a wider range of host plants possess more efficient mechanisms of biochemical adaptations to allelochemicals, and therefore oxidative stress can be generated to a lesser extent than in more specialized species [4].

The most common approach for controlling aphid populations is the application of synthetic insecticides. However, long-term and repeated use of these chemicals has led to the development of insecticide resistance in aphids, especially in *M. persicae* [5,6,7]. Consequently, it creates a cycle of increasing insecticide doses and growing insect resistance. Additionally, the residues associated with the use of pesticides are toxic to non-target organisms and hazardous to the environment. These problems have stimulated the research on alternative methods of pest control. Among other alternatives, the use of plant-derived products such as essential oils (EOs) has recently gained considerable attention. These botanicals are good sources of novel insect pest control agents because they contain many substances with active properties against insect pests including insecticides, repellents, antifeedants, ovicides and growth regulators [8,9,10,11]. The toxic properties of EOs were reported against aphids and other insect pests [12,13]. Moreover, EOs are typically complex mixtures that may act on multiple target sites and therefore the probability of developing a resistant population is very low [14,15].

An important aspect of efficient and safe use of EOs in plant protection is the elucidation of their mode of action towards the target pests. In general, EOs and their constituents exert their insecticidal activity through neurotoxic effects, such as interference with the neuromodulator octopamine or GABA-gated chloride channels, or by inhibition of acetylcholinesterase activity [14,16]. Some studies also suggest that the toxic effects of EOs may be related to their pro-oxidant activity [17,18,19]. However, exact mechanisms of this process have not yet been completely elucidated.

Previous studies have shown that different classes of allelochemicals can induce the generation of reactive oxygen species (ROS) within insect tissues [20,21]. An excessive amount of ROS results in oxidative stress that may lead to uncontrolled lipid peroxidation, protein oxidation and even cell death [22]. On the other hand, insects possess antioxidant defense mechanisms that help to combat the excess production of ROS and neutralize them. A portion of ROS is scavenged by dietary antioxidants, but most are eliminated by a suite of antioxidant enzymes, including superoxide dismutase (SOD) and catalase (CAT) [23].

Essential oils from Asteraceae plants possess a wide spectrum of biological activities, including antimicrobial, antifungal and insecticidal properties [9,24,25]. Our previous studies revealed that several EOs from these plants have potent insecticide activity against the green peach aphid [26]. The oils inhibited the activity of acetylcholinesterase and Na^+^/K^+^-ATPase, important enzymes of the insect nervous system, and upregulated the activity of glutathione S-transferase, a key detoxification enzyme. Based on the above results, we selected two EOs for further testing: the oil of *Santolina chamaecyparissus* L. which had the highest aphicidal activity and oil from *Tagetes patula* L. which had moderate activity. The current study was undertaken in order to deepen the knowledge of the biological and biochemical effects of selected EOs from Asteraceae plants on aphid species with different feeding adaptations, specifically the broad generalist *M. persicae* and the grass specialist *R. padi*. The specific objectives were: (i)Explore the effect of santolina and marigold EOs on population parameters of *M. persicae* and *R. padi*; (ii) Determine the levels of reactive oxygen species (ROS) (superoxide anion radical and hydrogen peroxide) and lipid peroxidation (expressed as concentration of thiobarbituric acid reactive substances, TBARS) within aphid tissues after treatment with EOs; and (iii) Analyze the changes in activity of the important antioxidant enzymes superoxide dismutase (SOD) and catalase (CAT) in both aphid species.

## 2. Materials and Methods

### 2.1. Aphid Culture

The aphids used in this study were obtained from a stock culture kept at the Siedlce University of Natural Sciences and Humanities, Poland. A parthenogenetic clone of *M. persicae* was reared on pea seedlings (*Pisum sativum* L.) whereas *R. padi* was reared on winter wheat (*Triticum aestivum* L.). Insect cultures were maintained in controlled environmental chambers at 23 ± 1 °C with 65% relative humidity (RH) and 16:8 h (L:D) photoperiod. Wingless adult parthenogenetic females in the first day of reproduction were selected for all subsequent experiments.

### 2.2. Plant Material and EO Extraction

EOs were extracted from the aerial parts of *S. chamaecyparissus* and *T. patula*, which are common ornamental plants from the Asteraceae family. The plant material was obtained from our own collection cultures located in Siedlce, Poland (52°17′ N, 22°24′ E, altitude 150 m, average annual rainfall 522 mm). The harvested material was dried in the shade at 25–30 °C and then powdered. Fifty grams of the plant material and 500 mL of water were subjected to water distillation for 3 h using a Clevenger-type apparatus. Anhydrous sodium sulphate was used to eliminate water from the extract. The obtained EOs were filtered and then stored in a sealed glass vials in a refrigerator (4 °C) until further use.

### 2.3. Bioassays

The bioassays were performed following the methodology previously described by Czerniewicz et al. [26]. The tested EOs were dissolved in ethanol to give stock solutions of 40% (*w*/*v*). The EOs solutions for bioassays were prepared by appropriate dilution of the stock solution in water with the addition of Tween 80 (0.075% *v*/*v*) as an emulsifier and ethanol (2% *v*/*v*). A mixture of Tween 80 and ethanol in water at the same concentration as above, but without the tested EOs, was used as a control solution. All bioassays were performed in controlled conditions at 23 ± 1 °C, 65% r.h. and L16:D8 photoperiod.

#### 2.3.1. Contact Toxicity Bioassays

Five concentrations of the tested EOs (0.1%, 0.2%, 0.4%, 0.6% and 0.8%) were used for toxicity tests and estimation of LC_50_ values. Fresh leaves from a suitable host plant (pea for *M. persicae* and wheat for *R. padi*) were placed in Petri dishes (20 cm diameter) lined with humid filter paper. Then, 20 aphid females were transferred to the leaves and allowed to settle for two hours. After settlement, the aphids were sprayed with aqueous emulsions of EOs or with a control solution. The solutions were applied with a laboratory sprayer at a rate of 1.5 mL per leaf with aphids. Ten replications were performed for all controls and treatments. Mortality was assessed after 24 h of exposure and LC_50_ values were calculated using probit analysis. The aphids were considered dead when no movement was observed after gentle prodding with a fine brush.

#### 2.3.2. Effects of EOs on Aphid Population Parameters

To evaluate the effects of EOs on population parameters of *M. persicae* and *R. padi*, the concentration of 0.2% was used. The applied concentration was selected based on the results of the contact toxicity bioassays, where it caused 10–35% aphid mortality. The methodology presented here was previously described by Chrzanowski et al. [27]. An adult apterous female of *M. persicae* or *R. padi* was placed on seven-day-old seedlings of pea or wheat, respectively. A single aphid was isolated using a transparent plastic cylinder (Ø 10 cm, height 30 cm) covered with muslin to permit ventilation and left to produce nymphs overnight. Five nymphs were then left on each seedling, whereas the other offspring and adults were removed. The two-day-old nymphs were sprayed with a 0.2% aqueous emulsion of the studied EO or with a control solution, prepared as described above. The individuals were observed daily to determine the duration of pre-reproductive period (PRP). When the aphids reached reproductive maturity, spraying was repeated. The surviving adults were monitored daily for a period equal to PRP, on each occasion recording the number of newborn nymphs. During these observations, all nymphs were removed from the seedlings after having been counted. The daily fecundity (DF) was expressed as the number of nymphs per living adult female per day. The intrinsic rate of natural increase (r_m_) was calculated using the Wyatt and White equation [28]:

r_m_ = 0.738 × (ln Md) × d^−1^

where d is the length of the PRP; Md is the number of nymphs produced in a reproductive period equal to d; 0.783 is the correction factor. This experiment was performed in 15 independent replicates for each aphid species for both the control and treated groups.

### 2.4. Insect Treatment and Biochemical Analyses

Approximately 300 apterous females of *M. persicae* or *R. padi* were caged on pea or wheat seedlings, respectively, and after 2 h the aphids were sprayed with an aqueous emulsion of EO at a concentration of 0.2% (*w*/*v*) or with a control solution, prepared as described above. The effect of the tested EOs on the level of oxidative stress markers and enzyme activities within aphid tissues was checked 12 h, 24 h, 48 h and 72 h after exposure. Collected aphids (50 individuals per replicate) were homogenized in 1 mL of ice-cold 50 mM potassium phosphate buffer pH 7.0 for the superoxide anion (O_2_^•−^), hydrogen peroxide (H_2_O_2_) and CAT assays; 1% phosphoric acid for the TBARS assay; or in 50 mM potassium phosphate buffer pH 7.8 for the SOD assay. The obtained homogenates were filtered and then centrifuged at 10,000× *g* for 20 min at 4 °C, as previously described by Łukasik and Goławska [20]. The supernatants were used for further analyses. All treatments and biochemical assays were replicated independently at least three times.

#### 2.4.1. Superoxide Assay

Superoxide anion content was determined using the nitro blue tetrazolium (NBT), as described by Green and Hill [29]. The reaction mixture was prepared by adding 0.5 mL of aphid homogenate to 0.5 mL NBT in 0.2 M phosphate buffer pH 7.8. The changes in absorbance were measured against the blank samples (without NBT) at a wavelength of 490 nm. The reduced activity of NBT by the aphid homogenates was expressed as ΔA_490_ × min^−1^ × mg^−1^ of protein.

#### 2.4.2. Hydrogen Peroxide Assay

The content of hydrogen peroxide was determined according to Green and Hill [29]. Briefly, 0.3 mL of aphid homogenate was added to 1 mL of reagent (4 mM 4-aminoantipyrine, 24 mM phenol, and 0.4 U/mL of peroxidase dissolved in 0.1 M phosphate buffer, pH 7.0) and the mixture was incubated at 25 °C for 10 min. The absorbance was measured at 510 nm against the blank, which contained extraction buffer instead of the aphid homogenate. The hydrogen peroxide content was calculated from a standard curve prepared with the respective concentrations of H_2_O_2_ and was expressed in nmol × mg^−1^ of protein.

#### 2.4.3. TBARS Assay

The concentration of TBARS was evaluated using the procedure described by Halliwell and Gutteridge [30] with some modification. The reaction mixture consisted of 1 mL of supernatant, 1 mL of 0.6% thiobarbituric acid (TBA) in 0.25 M HCl and 1 mL of 15% trichloroacetic acid (TCA). Simultaneously, two controls were prepared. The first one contained water instead of aphid homogenate, and in the second one TBA solution was replaced with water. The obtained mixtures were heated at 95 °C for 60 min, cooled in ice, and then centrifuged at 10,000× *g* for 20 min. The absorbance of supernatants was recorded at 535 nm and corrected by subtracting the absorbance of the second control from that obtained for the sample. The TBARS content was calculated using the extinction coefficient (ε = 156 mM^−1^ cm^−1^) and expressed as nmol × mg^−1^ of protein.

#### 2.4.4. SOD Assay

The activity of SOD was determined using the Beauchamp and Fridovich [31] method, based on inhibition of formazan formation from NBT in the presence of superoxide radical generators (xanthine and xanthine oxidase system). The reaction mixture comprised 1.0 mL of 50 mM potassium phosphate buffer pH 7.8, containing 0.15 mM EDTA, 0.1 mL of 7 mM xanthine, 0.1 mL of 0.25 mM NBT and 0.1 mL of enzyme extract. The obtained solution was then mixed with 0.1 mL of xanthine oxidase (0.2 U/mL) in order to start the reaction. Thereafter, change in absorbance was monitored for 5 min at 560 nm. One unit of SOD activity was defined as the amount of enzyme necessary to decrease the rate of NBT reduction to 50% and expressed as U × min^−1^ × mg^−1^ of protein.

#### 2.4.5. CAT Assay

CAT activity was determined according to the Aebi [32] method. For this purpose, 0.1 mL of the aphid homogenate was mixed with 0.1 mL of 30 mM H_2_O_2_ and 0.8 mL extraction buffer. The degradation of H_2_O_2_ was monitored at 240 nm for 3 min. The enzyme activity was calculated using molar extinction coefficient (ε = 43.6 M^−1^ cm^−1^), and the results were expressed as μmol of decomposed H_2_O_2_ × min^−1^ × mg^−1^ of protein.

#### 2.4.6. Protein Content

The protein concentration in the aphid homogenates was determined by the Bradford method [33]. The absorbance was measured at 595 nm using a microplate reader (BioTek, Winooski, VT, USA). Bovine serum albumin (BSA) was used as a protein standard.

### 2.5. Statistical Analysis

Probit analysis was conducted to estimate LC_50_ values with their corresponding 95% confidence limits (CL) by IBM SPSS v. 23 (IBM Corp., Armonk, NY, USA). LC_50_ values were considered significantly different when their respective 95% CLs did not overlap. The Kruskal–Wallis test (a nonparametric equivalent of ANOVA) was used to calculate differences in the population parameters of *M. persicae* and *R. padi* after treatment with EOs. Differences in the levels of oxidative stress markers were analyzed using a two-way ANOVA with EO and time of exposure as fixed effects. Analyses were performed separately for each aphid species. The significance of differences between mean values was calculated using Tukey’s multiple comparison post hoc test at *p* < 0.05. The calculations were carried out using Statistica v. 13.3 software (Statsoft, Poland).

## 3. Results

### 3.1. Toxicity of EOs towards M. persicae and R. padi

The tested EOs showed significant toxicity against adult females of *M. persicae* and *R. padi* after 24 h exposure (Table 1). Probit analysis showed that EO from *S. chamaecyparissus* exhibited a higher level of contact toxicity to both aphid species (LC_50_ = 0.25% for *R. padi* and LC_50_ = 0.34% for *M. persicae*) than EO obtained from *T. patula*, with corresponding LC_50_ values of 0.31% and 0.61%. Moreover, the results indicated that the tested EOs were less toxic to the green peach aphid than to the bird cherry-oat aphid females.

### 3.2. Effect of EOs on Biological Parameters of Aphids

The results showed that treatment with *S. chamaecyparissus* and *T. patula* EOs affected the development of both aphid species (Table 2). The PRP of *M. persicae* was prolonged by 0.65–0.71 days after treatment with the tested EOs, while in *R. padi* only EO from *T. patula* significantly prolonged the PRP (0.57 days) compared with the control. Moreover, a significant decrease in production of larvae by EO exposed apterous females for both aphid species was observed. Exposure of *M. persicae* to EOs from *S. chamaecyparissus* and *T. patula* limited daily fecundity by 49.9% and 41.3%, respectively. Application of the tested EOs to *R. padi* females reduced daily fecundity by 59.8% for the EO of *S. chamaecyparissus* and 33.1% for the EO of *T. patula*. When comparing the activity of the oils, it was shown that the EO from *S. chamaecyparissus* was more effective in decreasing aphids’ fecundity than the EO from *T. patula*, especially in *R. padi*. Treatment with EOs also led to a significant reduction in the intrinsic rate of natural increase in both aphid species, and similarly the stronger reduction was observed after application of the EO from *S. chamaecyparissus* (25–29%) than *T. patula* (16–22%).

### 3.3. Effect of EOs on the Level of Oxidative Stress Markers within Aphid Tissues

**Figure 1:**
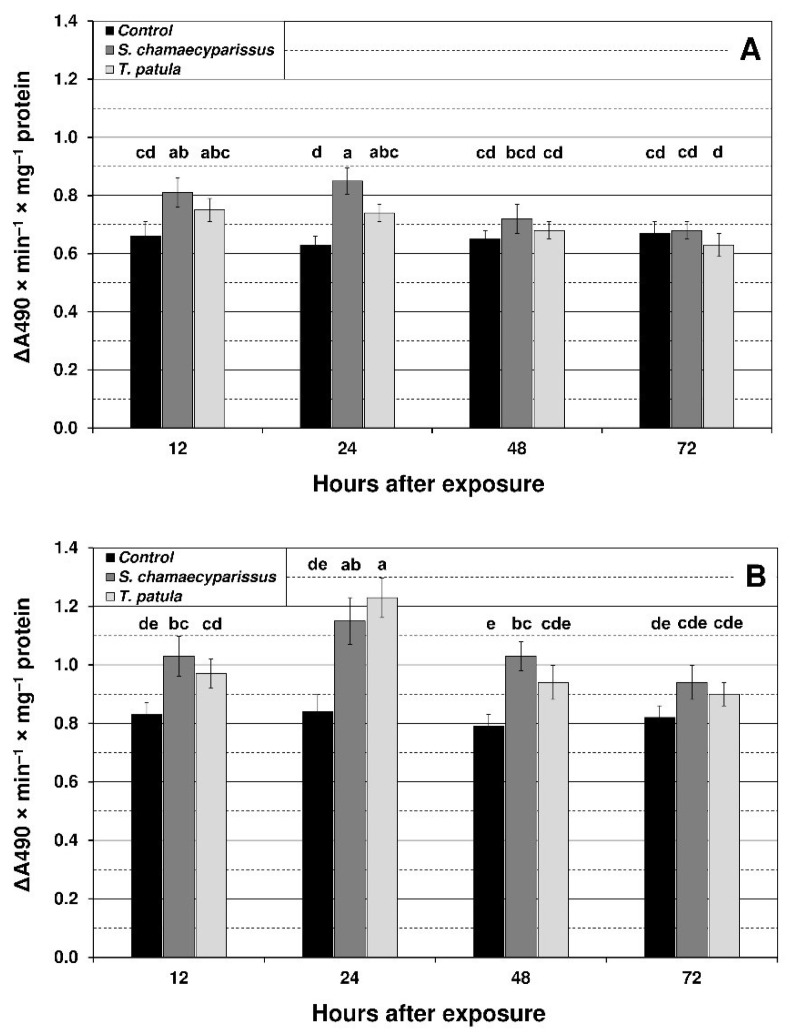
Effect of *S. chamaecyparissus* and *T. patula* essential oils on the concentration of superoxide anion (mean ± SD) within tissues of *M. persicae* (**A**) and *R. padi* (**B**). Values marked by different letters are significantly different at *p* < 0.05 (Tukey’s test). Statistics for two-way ANOVA: (**A**) treatment F_2,24_ = 22.99, *p* < 0.001, time F_3,24_ = 9.41, *p* < 0.001, treatment × time interaction F_6,24_ = 4.21, *p* < 0.01; (**B**) treatment F_2,24_ = 51.27, *p* < 0.001, time F_3,24_ = 18.17, *p* < 0.001, treatment × time interaction F_6,24_ = 4.82, *p* < 0.01.

**Figure 2:**
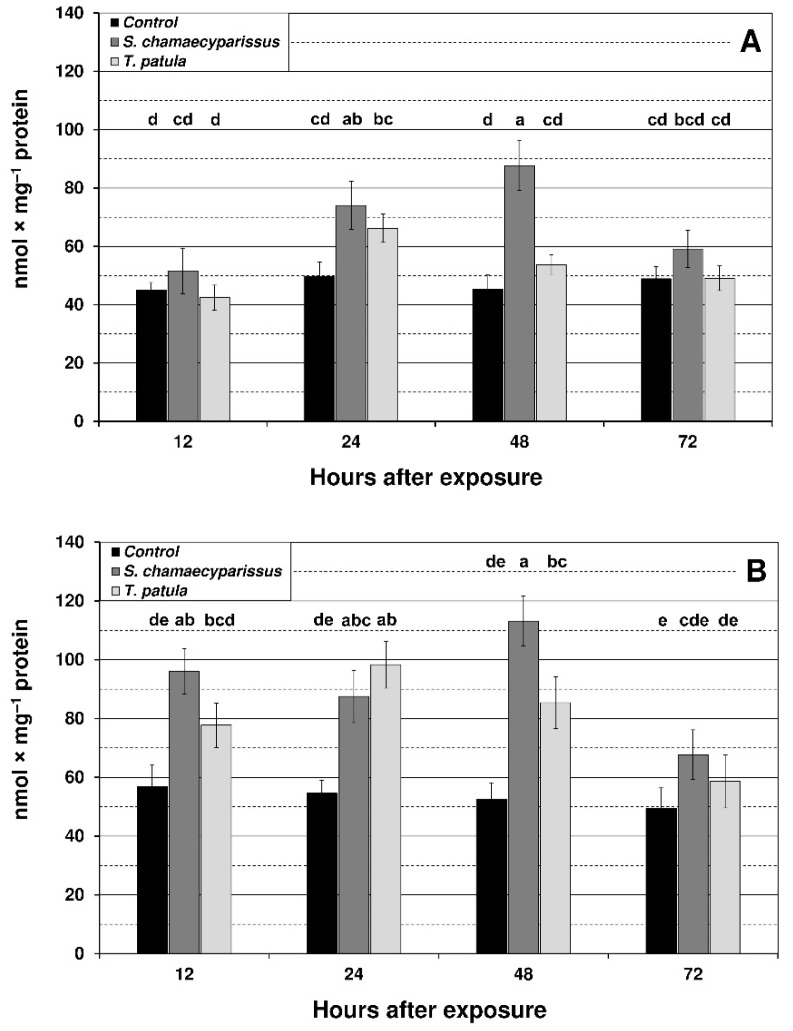
Effect of *S. chamaecyparissus* and *T. patula* essential oils on the concentration of hydrogen peroxide (mean ± SD) within tissues of *M. persicae* (**A**) and *R. padi* (**B**). Values marked by different letters are significantly different at *p* < 0.05 (Tukey’s test). Statistics for two-way ANOVA: (**A**) treatment F_2,24_ = 37.45, *p* < 0.001, time F_3,24_ = 13.11, *p* < 0.001, treatment × time interaction F_6,24_ = 7.17, *p* < 0.001; (**B**) treatment F_2,24_ = 59.49, *p* < 0.001, time F_3,24_ = 14.76, *p* < 0.001, treatment × time interaction F_6,24_ = 5.29, *p* < 0.01.

**Figure 3:**
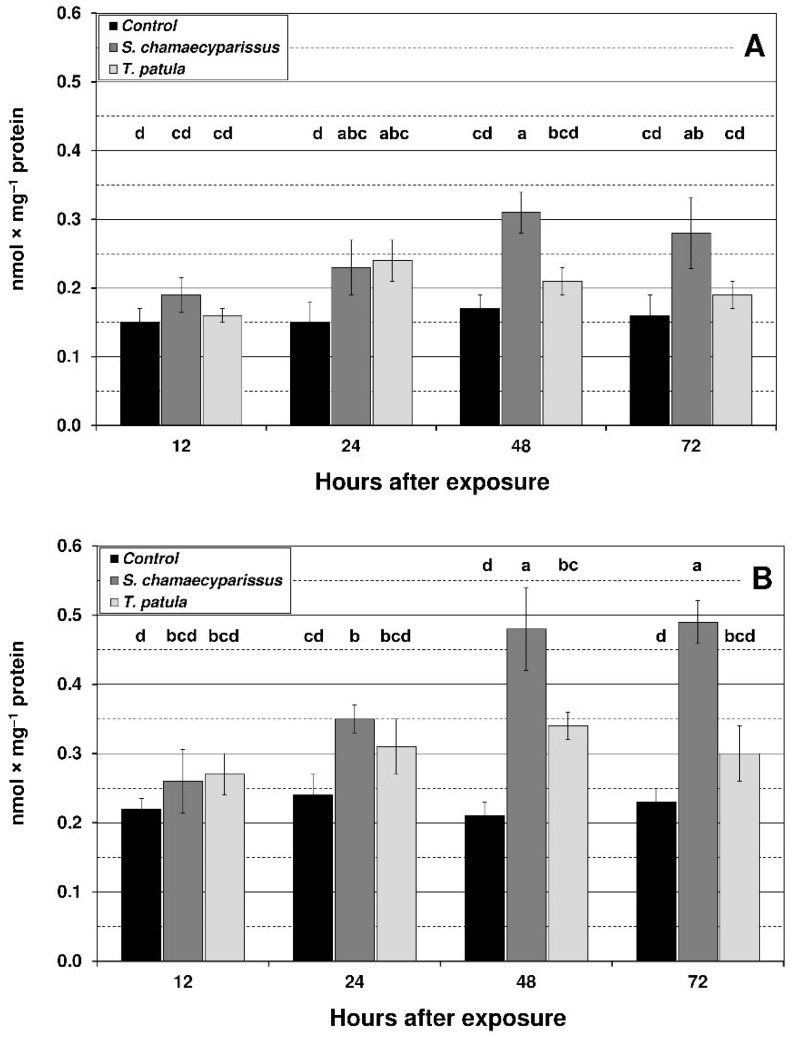
Effect of *S. chamaecyparissus* and *T. patula* essential oils on the concentration of lipid peroxidation products (TBARS) (mean ± SD) within tissues of *M. persicae* (**A**) and *R. padi* (**B**). Values marked by different letters are significantly different at *p* < 0.05 (Tukey’s test). Statistics for two-way ANOVA: (A) treatment F_2,24_ = 31.12, *p* < 0.001, time F_3,24_ = 7.63, *p* < 0.001, treatment × time interaction F_6,24_ = 4.55, *p* < 0.01; (B) treatment F_2,24_ = 73.50, *p* < 0.001, time F_3,24_ = 15.15, *p* < 0.001, treatment × time interaction F_6,24_ = 9.56, *p* < 0.001.

Results showed that treatment with EOs from *S. chamaecyparissus* and *T. patula* led to significant changes in the level of oxidative stress markers within tissues of *M. persicae* and *R. padi*. The content of superoxide anion, hydrogen peroxide and TBARS was modified by treatment, time and the treatment × time interaction (Figure 1, Figure 2 and Figure 3). The concentration of superoxide anion in the green peach aphid significantly increased in comparison to the control at 12 h after application of *S. chamaecyparissus* EO, and only at 24 h after treatment with EO from *T. patula* (Figure 1). In the bird cherry-oat aphid, the level of this compound was significantly higher at 12 h, 24 h and 48 h for *S. chamaecyparissus* EO, and at 24 h for *T. patula* EO. The observed increase in superoxide anion concentration was more pronounced in *R. padi* (37–46%) than in *M. persicae* (17–35%). Finally, at 48 h and 72 h in *M. persicae* and at 72 h in *R. padi*, the superoxide anion concentration flattened and was similar to untreated insects.

Levels of hydrogen peroxide in the green peach aphid significantly increased at 24 h and 48 h after application of *S. chamaecyparissus* EO (Figure 2). For the bird cherry-oat aphid tissues, a significant increase in hydrogen peroxide generation was observed at 12 h after treatment with EO from *S. chamaecyparissus*, and at 24 h and 48 h for both of the applied EOs. The highest increase for *S. chamaecyparissus* EO was noted after 48 h of exposure (115%) and for *T. patula* EO after 24 h of exposure (80%). It is also worth noting that the observed increase in hydrogen peroxide content was much higher in *R. padi* than in *M. persicae*.

The content of TBARS showed an upward tendency in both aphid species after exposure to the tested EOs (Figure 3). The highest level of TBARS was shown at 48 and 72 h after treatment with EO from *S. chamaecyparissus*. Compared to the control, the increase in *R. padi* was more noticeable than in *M. persicae,* i.e., in *R. padi* at 48 h and 72 h it was approximately 100%, and in *M. persicae* by approximately 70%. Treatment with EO from *T. patula* also led to an increase in the content of TBARS; however, this increase was lower in comparison to EO from *S. chamaecyparissus*, and significant changes were only found in *M. persicae* after 24 h exposure and in *R. padi* after 48 h exposure (approximately 60% increase in both cases).

### 3.4. Effect of S. chamaecyparissus and T. patula EOs on Activity of Antioxidant Enzymes in Aphids

**Figure 4:**
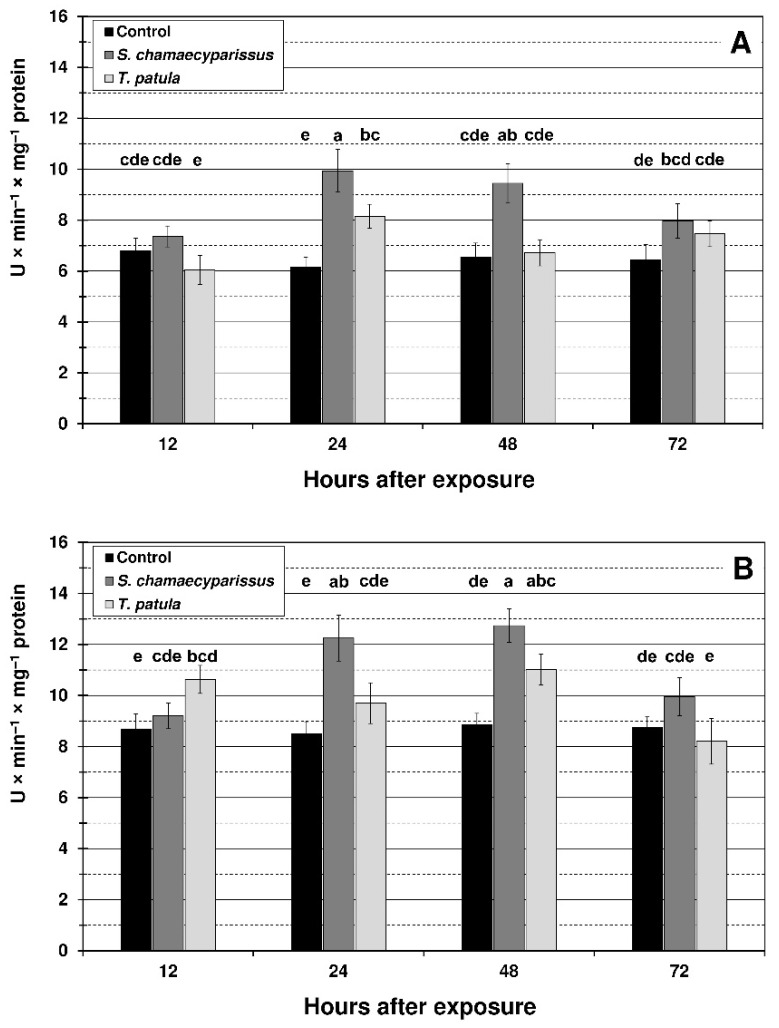
Activity of superoxide dismutase (mean ± SD) in apterous females of *M. persicae* (**A**) and *R. padi* (**B**) after treatment with selected Asteraceae essential oils. Values marked by different letters are significantly different at *p* < 0.05 (Tukey’s test). Statistics for two-way ANOVA: (**A**) treatment F_2,24_ = 49.66, *p* < 0.001, time F_3,24_ = 9.19, *p* < 0.001, treatment × time interaction F_6,24_ = 6.92, *p* < 0.001; (**B**) treatment F_2,24_ = 38.57, *p* < 0.001, time F_3,24_ = 14.27, *p* < 0.001, treatment × time interaction F_6,24_ = 8.91, *p* < 0.001.

**Figure 5:**
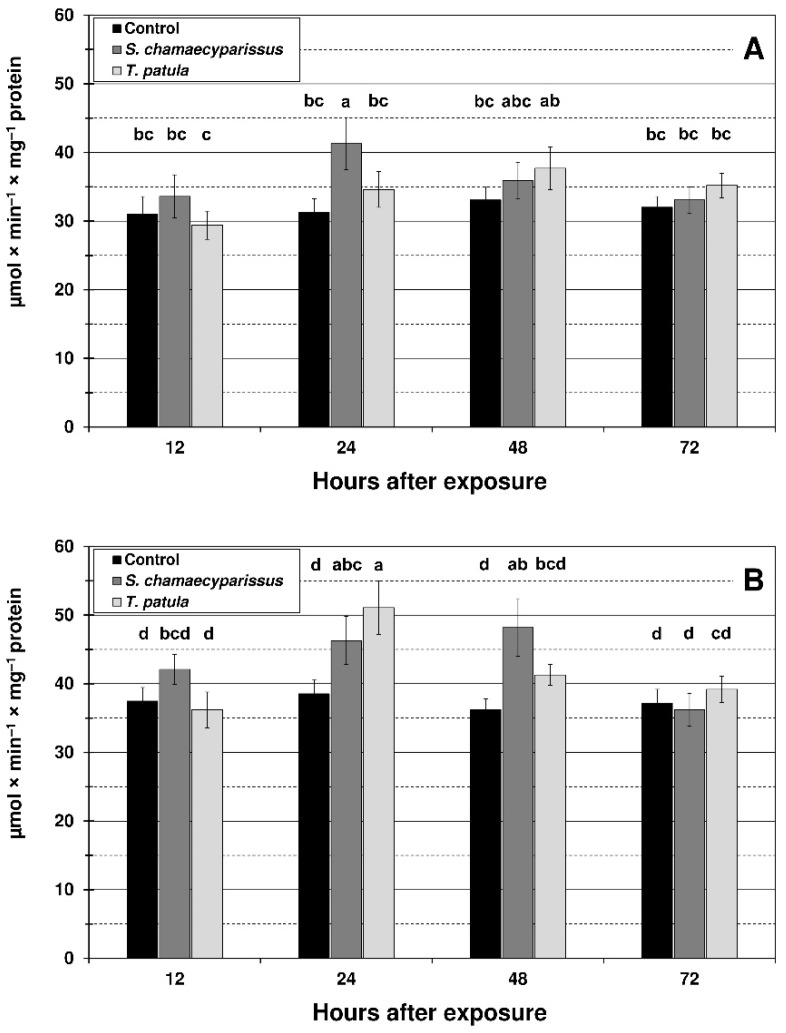
Activity of catalase (mean ± SD) in apterous females of *M. persicae* (**A**) and *R. padi* (**B**) after treatment with selected Asteraceae essential oils. Values marked by different letters are significantly different at *p* < 0.05 (Tukey’s test). Statistics for two-way ANOVA: (**A**) treatment F_2,24_ = 8.19, *p* < 0.001, time F_3,24_ = 6.10, *p* < 0.01, treatment × time interaction F_6,24_ = 3.43, *p* < 0.01; (**B**) treatment F_2,24_ = 16.49, *p* < 0.001, time F_3,24_ = 16.07, *p* < 0.001, treatment × time interaction F_6,24_ = 7.26, *p* < 0.001.

Treatment with the tested EOs affected the activity of SOD and CAT within tissues of the green peach aphid and the bird cherry-oat aphid. Activities of the studied antioxidant enzymes changed with treatment, time and the treatment × time interaction (Figure 4 and Figure 5). EO from *S. chamaecyparissus* significantly increased the activity of SOD in both aphid species after 24 and 48 h of exposure (Figure 4). The application of *T. patula* EO significantly increased the SOD activity at 24 h in *M. persicae*, and at 12 h and 48 h in *R. padi*. When comparing differences between the oils, stronger induction in the activity of superoxide dismutase within aphid tissues was generally shown after treatment with the EO from *S. chamaecyparissus*.

The activity of CAT in *M. persicae* significantly increased in comparison with the control only at 24 h after treatment with EO from *S. chamaecyparissus* (Figure 5). After spraying the aphid with *T. patula* EO, an increase in CAT activity was observed at most of the studied time intervals, however the changes were not statistically significant. In the bird cherry-oat aphid, the activity of CAT was significantly elevated at 24 h and 48 h after application of *S. chamaecyparissus* EO, and at 24 h after treatment with the EO of *T. patula*. It is worth noting that the activity of SOD and especially CAT in treated insects was similar to the control level after 72 h of exposure.

## 4. Discussion

EOs are complex mixtures of secondary plant metabolites. They generally consist of monoterpenes, sesquiterpenes and biogenetically related phenols, all of which are characterized by low molecular weight [24,34]. These mixtures of metabolites interfere with insect physiology through different mechanisms and at various target sites. In our previous research [26], the composition of the studied EOs was chemically characterized by GC-MS analysis. It was shown that the EO extracted from *S. chamaecyparissus* mainly consisted of artemisia ketone (25.9%), *β*-phellandrene (18.7%), vulgarone B (11.6%) and *β*-myrcene (9.2%), while the main components of *T. patula* EO were terpinolene (15.8%), limonene (12.5%) and piperitone (9.8%).

The results of this study revealed that the tested EOs exhibit potent contact toxicity against the green peach aphid and the bird cherry-oat aphid. The values analyzed from LC_50_ spraying bioassays showed that the EO from *S. chamaecyparissus* was more toxic than that from *T. patula* in both aphid species. This finding could be related to the structural and biological activity of the constituents of the EOs. The bioactivity of EOs and other extracts from different *Tagetes* species have been previously reported with reference to several insect pests, including aphids [35,36,37]. Dardouri el al. [38] demonstrated that volatiles from *T. patula* significantly reduced performance of the green peach aphid on pepper. Essential oil and extracts from *Tagetes minuta* were also toxic to *M. persicae* and two other aphid species—*Acyrthosiphon pisum* (Harris) and *Aulacorthum solani* (Kaltenbach) [36]. There is less data available on the insecticidal properties of santolina oil; however, with regard to aphids, some studies revealed the oil had strong antifeedant activity against *R. padi* [39]. Moreover, in contact toxicity tests it was shown that essential oil from santolina exhibited potent contact toxicity towards *Reticulitermes speratus* and *Blattella germanica* [40,41]. These studies revealed that the toxicity of the oil was strongly connected with its main components, such as *β*-phellandrene and artemisia ketone. In addition, it was shown that the insecticidal activity of *β*-phellandrene correlated with its ability to inhibit the activity of acetylcholinesterase. In general, the most abundant constituents of EOs determine their biological activity [42,43]. Previous studies have demonstrated that the main components of *T. patula* oil also possess insecticidal properties. Tomova et al. [36] reported relatively high toxicity of limonene to the pea aphid. Moreover, in studies on fumigant toxicity of nine monoterpenes against *M. persicae*, it was shown that terpinolene was one of the most toxic, next to carvacrol and bornyl acetate [44].

For effective pest management, not only direct mortality of adults, but also limitation of population parameters is important. The results reported here show that application of the EOs from *S. chamaecyparissus* and *T. patula* even at sublethal concentration negatively affects the bionomic parameters of *M. persicae* and *R. padi*. Spraying with the EOs significantly reduced daily fecundity of aphid females and led to a decrease in the intrinsic rate of natural increase. Moreover, the PRP was prolonged by about 10% compared with untreated insects. Previous studies demonstrated that several EOs possess properties similar to juvenile hormones and act as insect growth regulators [45,46]. They induced disruption in growth and the reproduction of insects. Sammour et al. [45] showed that treatment with basil oil caused prolongation of the nymphal development of *Aphis craccivora*. Moreover, Chopa and Descamps [47] reported reduction of the net reproductive rate and the intrinsic rate of population increase in *Metopolophium dirhodum* after treatment with EOs from *Schinus areira* and *Tagetes terniflora*. The results presented here indicate that the bird cherry-oat aphid is more sensitive to the tested EOs than the green peach aphid. This was confirmed in both toxicity bioassays and analysis of biological parameters. Such variation in aphid response may be due to the different biochemical and physiological adaptations to herbivory. Generally, specialists show a greater sensitivity to allelochemicals and react at lower concentrations than generalists [48]. Łukasik et al. [4] showed that the monophagous grain aphid *Sitobion avenae* F. after exposure to phenolic compounds had significantly higher level of oxidative stress markers than the oligophagous *R. padi*. On the other hand, Bruce et al. [49] showed that *M. persicae* was less sensitive to the *Hemizygia petiolata* essential oil treatment than *S. avenae* and *A. pisum*. Moreover, our earlier studies indicated a relatively low level of *M. persicae* sensitivity towards plant secondary metabolites during research on aphicidal activity of phenolic-rich extracts obtained from several medicinal plants [50].

The present results revealed that treatment with EOs causes significant increases in levels of O_2_^•−^ and H_2_O_2_ within aphid tissues. The formation of superoxide anion may initiate a cascade of free radical reactions that generate other ROS including highly reactive hydroxyl radical (HO^•^). Excessive accumulation of ROS leads to disordered redox balance in an organism, inflicting serious damage to biological macromolecules, such as DNA, RNA, proteins and lipids. Hydroxyl radical is the main factor in free-radical toxicity because it is able to initiate lipid peroxidation. Lipid hydroperoxides may undergo secondary reactions producing highly reactive aldehydes and ketones called TBARS [4,51]. Our results showed an increased level of TBARS in both aphid species after treatment with the tested EOs, indicating that lipid peroxidation occurred within their tissues. Interestingly, the oil of santolina had both a higher aphicidal activity and evoked a higher level of lipid peroxidation. The effect was especially pronounced at 48 and 72 h post-exposure. These findings suggest that oxidative damage may be an important factor contributing to the insecticidal activity of the tested EOs, and therefore may be one of the mechanisms responsible for their toxic effects. Similar observations have been reported in earlier studies on the toxicity mechanisms of *Psidium guajava* (Myrtaceae) EO towards *Drosophila melanogaster* [52]. The studies presented evidence of oxidative stress, including ROS and TBARS formation, as well as changes in an important antioxidant response system. Shahriari et al. [19] also demonstrated a significant increase in lipid peroxidation products in *Ephestia kuehniella* (Lepidoptera) larvae after 24 and 48 h of feeding on a diet containing EO constituents such as α-pinene, trans-anethole and thymol. For phytophagous insects, lipid peroxidation is especially harmful because it leads to disruption of cell membrane permeability and many other physiological functions such as development and reproduction [53].

Aphids and other herbivorous insects possess an antioxidant enzyme system that scavenges excessive ROS. The SOD-CAT system provides the first defense against oxygen toxicity by catalyzing the dismutation of superoxide anion to hydrogen peroxide and decomposition of hydrogen peroxide to water and molecular oxygen [20,54]. Our results demonstrate that treatment with EOs leads to a significant upregulation of SOD and CAT activity in both aphid species. This upregulation of antioxidant enzymes could be an adaptive response in insects, aimed at minimalizing potentially deleterious effects of the EO-induced oxidative stress. The enzyme activity increased markedly after 24 or 48 h of exposure to EOs and in most cases returned to the control level after 72 h. Significant induction of SOD and CAT activity at 24 and 48 h was also reported by Shahriari et al. [19] in *E. kuehniella* after feeding on a diet containing several EO components. On the other hand, comparative studies of antioxidant enzyme activity in two stored product pests exposed to *Rosmarinus officinalis* EO showed that, in *Sitophilus oryzae,* SOD and CAT activity increased after 48 h, while in *Oryzaephilus surinamensis* it increased after only 6 h [15]. In studies on the mechanism of toxicity of *Eugenia uniflora* EO towards fruit flies, Cunha et al. [21] described a two-phased adaptive response to oxidative stress. This consisted of an early phase triggered by ROS induction, resulting in activation of the master regulator of the cellular antioxidant response (the transcription factor Nrf2), and a late phase, characterized by oxidative damage and increased levels of ROS and xenobiotic detoxifying enzymes. However, it should be noted that differences in the antioxidant response of insects to oxidative stress may depend on factors such as the applied compound and its concentration, type of treatment, insect genus and time of exposure [55,56,57].

Comparing the biochemical response of the studied aphid species under treatment with EOs, it was shown that the level of oxidative stress markers was distinctly higher in *R. padi* than in *M. persicae*. The relatively low level of oxidative stress in the green peach aphid may result from the very efficient mechanisms of xenobiotic detoxification in this species. Castells and Berenbaum [58] and Wen et al. [59] suggest that polyphagous insects possess an expanded enzymatic system able to remove a wide range of plant allelochemicals. Moreover, our previous studies [26], have shown upregulation of detoxification mechanisms based on glutathione S-transferase within the green peach aphid under Asteraceae EOs treatment. This enzyme belongs to the phase II detoxification system and catalyzes the reduced glutathione conjugation with activated (in phase I) toxic metabolites. Among phase I detoxification enzymes, the cytochrome P450 monooxygenases (P450s) have been assumed as crucial proteins that oxidize botanical pesticides [60]. However, further research is necessary to explain the role of P450s in detoxification of the essential oil components in the studied aphids.

## 5. Conclusions

These results show that EOs from *S. chamaecyparissus* and *T. patula* exhibit considerable toxicity against *M. persicae* and *R. padi*, significantly limiting the aphids’ biological parameters even at sublethal levels. The mode of action of these oils may be related to the induction of oxidative stress within aphid tissues, since increased levels of ROS and accumulation of lipid peroxidation products was noted after treatment with the tested EOs. In addition, the increased activity of important antioxidant enzymes indicates that an adaptive response to oxidative stress occurred in EO-exposed aphids. The oligophagous *R. padi* was more sensitive to the EO treatment than the highly polyphagous *M. persicae*. The relatively high levels of resistance in the green peach aphid are most likely related to its highly effective mechanisms of xenobiotic detoxification, however further research is needed to explain this phenomenon.

## Figures and Tables

**Table 1:** Toxicity of the studied essential oils to apterous females of *M. persicae* and *R. padi.*

Aphid Species	Essential Oil	LC_50_^a^	95% CL ^b^	Slope ± SE	χ^2^	*P*
*M. persicae*	*S. chamaecyparissus*	0.34	0.31−0.36	2.88 ± 0.22	29.29	0.843
	*T. patula*	0.61	0.56−0.67	2.67 ± 0.24	27.13	0.905
*R. padi*	*S. chamaecyparissus*	0.25	0.22−0.29	2.82 ± 0.18	27.63	0.884
	*T. patula*	0.31	0.27−0.35	2.73 ± 0.22	30.48	0.798

^a^ Lethal concentration (% *w*/*v*). ^b^ 95% confidence limit.

**Table 2:** Effect of *S. chamaecyparissus* and *T. patula* essential oils (at a concentration of 0.2%) on the population parameters of *M. persicae* and *R. padi*.

Aphid Species	Treatment	PRP	DF	r_m_
*M. persicae*	Control	7.76 ± 0.41 b	3.27 ± 0.43 bc	0.31 ± 0.02 c
	*S. chamaecyparissus*	8.47 ± 0.51 a	1.64 ± 0.56 d	0.22 ± 0.04 d
	*T. patula*	8.41 ± 0.43 a	1.92 ± 0.53 d	0.24 ± 0.03 d
*R. padi*	Control	5.96 ± 0.34 d	5.95 ± 0.84 a	0.44 ± 0.03 a
	*S. chamaecyparissus*	6.35 ± 0.38 cd	2.93 ± 0.87 c	0.33 ± 0.04 c
	*T. patula*	6.53 ± 0.46 c	3.98 ± 0.72 b	0.37 ± 0.03 b
H_(5, 90)_		73.82	70.74	78.26
p		<0.0001	<0.0001	<0.0001

PRP—pre-reproductive period (days); DF—daily fecundity per apterous adult during the period equal to PRP; r_m_—intrinsic rate of natural increase. Means (±SD) within columns followed by different letters are significantly different at *p* < 0.05 (Tukey’s test).

## Data Availability

Data is contained within the article.
